# Laser‐Assisted Mo_2_C‐Derived Patterned Oxide for Highly Selective Room Temperature Ammonia Sensor for Food Spoilage Monitoring

**DOI:** 10.1002/smtd.202501246

**Published:** 2025-09-16

**Authors:** Radha Bhardwaj, Sujit Deshmukh, Martin Pumera

**Affiliations:** ^1^ Future Energy and Innovation Laboratory Central European Institute of Technology Brno University of Technology Purkyňova 123 Brno 61200 Czech Republic; ^2^ Department of Medical Research China Medical University Hospital China Medical University No. 91 Hsueh‐Shih Road Taichung 40402 Taiwan; ^3^ Department of Chemical and Biomolecular Engineering Yonsei University 50 Yonsei‐ro, Seodaemun‐gu Seoul 03722 South Korea; ^4^ Faculty of Electrical Engineering and Computer Science VSB – Technical University of Ostrava 17. listopadu 2172/15 Ostrava 70800 Czech Republic

**Keywords:** food spoilage, laser engineered, Mo_2_C, NH_3_ sensor, selectivity

## Abstract

The need for advanced gas sensors has risen for the detection of hazardous gases, breath analysis, and food industry applications. Transition metal carbides (TMCs), like Mo_2_C, are novel gas‐sensing materials attributed to high electronic conductivity and superior catalytic properties. Poor sensitivity and selectivity are big concerns in TMC‐based sensors due to their low specific surface area and fewer reactive sites. Partial oxidation of Mo_2_C offers the tuning of structural, chemical, and electronic properties. However, conventional techniques, annealing, and solution processing offer uncontrolled oxidation and lead to structural degradation. Herein, by using a temporally and spatially controlled picosecond (ps) pulsed laser, micropatterned Mo_2_C‐derived oxide (MoO_3_) is developed at room temperature for highly efficient ammonia (NH_3_) sensing. The uniformly decorated MoO_3_ nanoclusters over Mo_2_C function as active centers for better NH_3_ interaction and formation of discrete Schottky barriers (SBs) between materials, tuning the charge carrier transportation. The MoO_3_/Mo_2_C sensor exhibited excellent selectivity toward NH_3_ over other interfering gases like hydrogen, ethanol, and acetone. This sensor showed excellent sensitivity (351%/100 parts per billion (ppb) NH_3_) and long‐term stability. The Mo_2_C laser‐treated sensor has been successfully tested for monitoring food spoilage. Laser‐assisted engineering will provide a new avenue for designing highly efficient gas sensors.

## Graphical abstract

Laser‐assisted Mo_2_C‐derived oxide sensors can be used for selective ammonia sensing at ambient conditions.

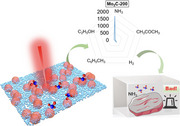

## 1 Introduction

The constant demand for gas sensors in the detection and monitoring of toxic gases for a variety of applications requires rapid advancement in gas sensing technology. Among various gases, NH_3_ has the great attention from researchers due to its wide use in the food industry, air quality monitoring, and breath analysis applications.^[^ ^1^, ^2^ ^]^ In food safety and quality monitoring, NH_3_ is an efficient biomarker for monitoring high‐protein food deterioration, such as fish and shrimp.^[^ ^1^, ^3^, ^4^ ^]^ Therefore, there is a need to design high‐performance NH_3_ sensors with high selectivity, strong sensitivity, and room temperature real‐time monitoring.^[^ ^3^ ^]^ Among various gas sensing technologies, chemiresistive sensors have attracted the most attention owing to their simplicity, low cost, and long lifespan.^[^ ^5^, ^6^ ^]^ However, due to the poor selectivity and high working temperature of established sensing technology based on metal oxides, limiting their future employment, the research community has lately focused on developing advanced, high‐performing NH_3_ gas sensors.^[^ ^7^, ^8^, ^9^ ^]^

The TMCs are emerging semiconducting materials that have gathered huge attention in different forms, like carbide nanoparticles,^[^ ^10^, ^11^, ^12^ ^]^ and 2D; MXenes^[^ ^13^, ^14^, ^15^, ^16^, ^17^ ^]^ as an efficient gas sensing material. TMCs, such as Mo_2_C, W_2_C, and NbC, form a large family of materials that possess metallic conductivity, a high signal‐to‐noise ratio, and good thermal and chemical stability.^[^ ^11^, ^12^, ^18^, ^19^, ^20^ ^]^ Atomically thin layers of transition metal carbides belong to a distinct class of 2D materials called MXenes, which have been widely explored for the detection of gases or volatile organic compounds (VOCs) at room temperature.^[^ ^14^, ^15^, ^16^, ^17^ ^]^ Carburized TMCs, such as Mo_2_C nanoparticles, exhibit greater resistance to oxidation and superior ambient stability compared to 2D Mo_2_C MXene; however, they suffer from a limited specific surface area and restricted gaseous adsorption or reactive sites.^[^ ^10^, ^12^, ^14^, ^16^ ^]^ Nevertheless, unprecedentedly high signal‐to‐noise ratio (SNR) and superior catalytic activities of carburized Mo_2_C provide the ability to detect the ppb levels of NH_3_ and other gases and require further engineering to use them more effectively as highly selective sensing materials.^[^ ^10^, ^11^ ^]^ In particular, carburized Mo_2_C and their nanocomposite sensors have shown good sensitivity toward gases such as NO_2_,^[^ ^10^ ^]^ NH_3_,^[^ ^11^ ^]^ toluene,^[^ ^12^ ^]^ and acetone,^[^ ^12^ ^]^ at room temperature.

Heterointerface‐engineered nanomaterials demonstrate superior gas sensing properties compared to pristine materials due to enhanced specific surface area, surface reactive sites, and modulated charge transport in discrete Schottky heterojunctions.^[^ ^11^, ^12^, ^21^, ^22^, ^23^ ^]^ Choosing an appropriate engineering route is crucial and can significantly impact the gas sensing characteristics, like sensitivity, selectivity, response time, and stability.^[^ ^11^, ^12^, ^24^, ^25^ ^]^ A common way of heterointerface engineering involves the partial oxidation of the sensing materials, which has garnered interest for enhancing selectivity and offering improved structural stability and uniformity in the nanomaterial.^[^ ^25^, ^26^, ^27^, ^28^ ^]^ Thus far, several methods such as annealing at an elevated temperature,^[^ ^27^, ^29^, ^30^ ^]^ chemical process,^[^ ^26^, ^31^, ^32^, ^33^, ^34^ ^]^ hydrothermal,^[^ ^31^, ^33^ ^]^ and microwave plasma^[^ ^35^ ^]^ have been reported to oxidize the 2D TMC materials for improved gas sensing results. The oxidation transforms the metallic TMC materials into the counter semiconductor oxide, which modulates the Schottky barrier of the heterojunction by manipulating active sites.^[^ ^30^, ^33^ ^]^ However, the precise control of the degree of oxidation during these treatments is quite challenging. For example, the most common, traditional thermal annealing approach not only oxidizes the surface but also changes the microstructures, resulting in degradation of device performance.^[^ ^27^ ^]^ On the other hand, advanced controlled oxidation techniques, such as the multiphoton‐induced femtosecond laser (fs) method, can directly manipulate the oxygen content with a high resolution (resolution: ∼1 µm) in environmental conditions at room temperature.^[^ ^12^, ^20^, ^25^, ^36^ ^]^ This technique offers high control over oxidation, preventing structural degradation, and strong integration between the oxide and the TMC material.^[^ ^12^, ^36^ ^]^ Till now, laser‐assisted direct oxidation has been implemented in applications such as Li/Zn‐ion Batteries, supercapacitors,^[^ ^25^, ^37^ ^]^ and electronics^[^ ^20^ ^]^ and can be implemented in the designing of TMC‐derived oxide‐based heterostructure devices for efficient gas sensing characteristics at ambient conditions.

In this work, for the first time, we reported a single‐step temporally and spatially controlled pulsed laser‐induced patterned oxidation in carburized Mo_2_C at room temperature and ambient conditions (63 ± 2% relative humidity (RH)) for enhanced NH_3_ sensing performance. A laser with varying power from 100 to 300 mW was used to initiate the oxidation of Mo_2_C without hampering the nanostructure of the Mo_2_C material. After absorbing the laser infrared energy, the Mo_2_C surface partially oxidizes MoO_3_ nanoclusters and forms a stable heterointerface, which consequently modulates the Schottky barrier and increases the active sites. Additionally, to demonstrate the practical applicability of the sensor food quality monitoring application, the fabricated device was placed on an open gas sensing platform capable of fish spoilage monitoring at ambient conditions. A gas sensing mechanism was established to understand the role of MoO_3_/ Mo_2_C heterostructure on the selective sensing of NH_3_ over other gases and VOCs. Moreover, by controlling the exposed surface‐active sites with the help of laser‐induced patterned partial oxidation, we can effectively tailor the gas sensitivity and selectivity properties of the TMC materials. This study further explains that precise incorporation of metal oxide (MoO_3_) in a regulated manner can enhance gas sensing capabilities, even at ambient temperatures, aiding in the development of advanced gas sensors for food spoilage monitoring applications.

## 2 Results and Discussion

**Figure 1:**
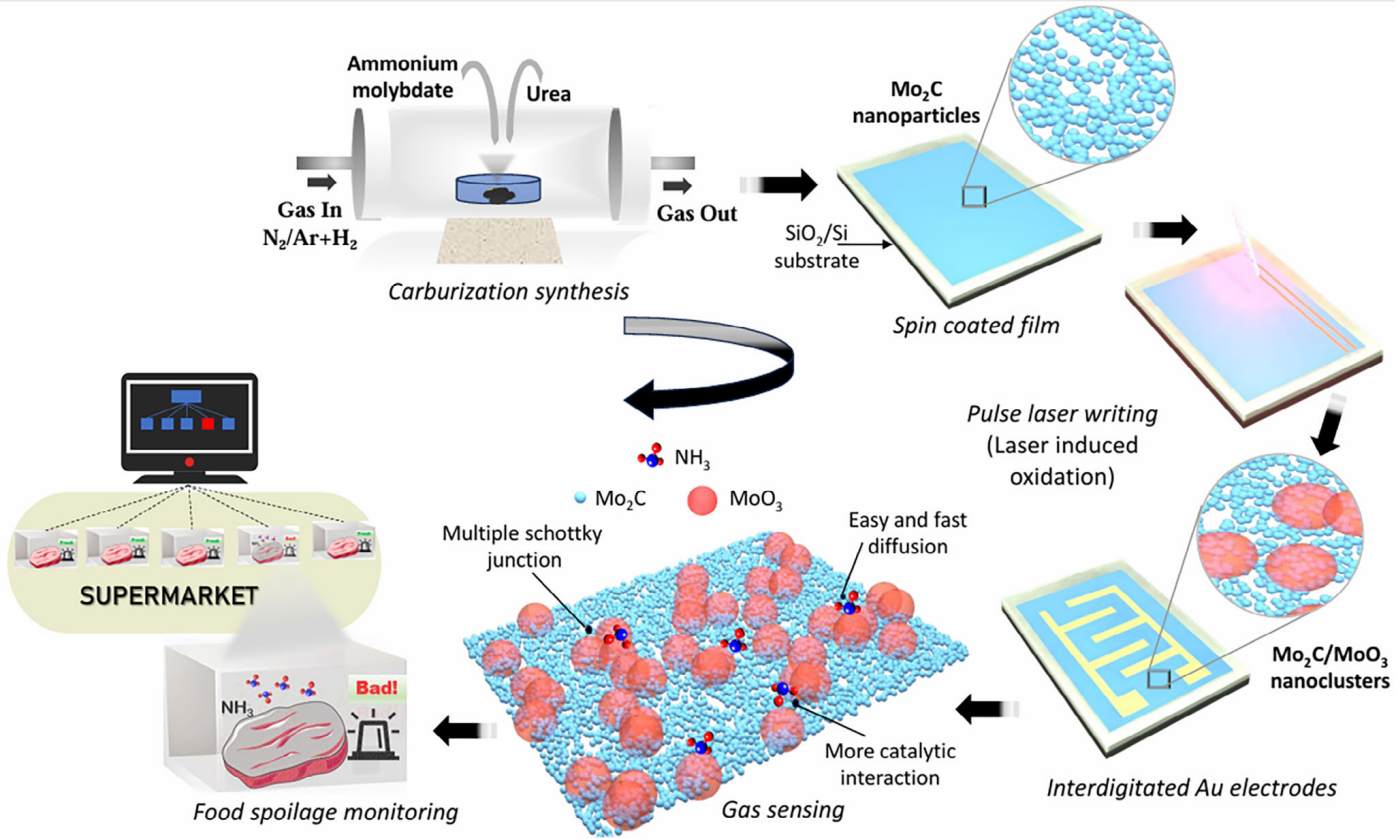
Schematic illustration of the fabrication procedure of laser‐induced Mo_2_C‐derived patterned oxide (MoO_3_) heterostructured NH_3_ sensor for food quality monitoring application.

**Figure** **1** illustrates the workflow schematic, which contains the fabrication procedure and its application scenarios. First, Mo_2_C nanoparticles were synthesized through the carburization process. A thin layer of the synthesized nanoparticles was then spin‐coated (500 rpm 2 min^−1^) on the SiO_2_/Si substrate. The dried thin film underwent the pulse laser writing process with a laser wavelength of 532 nm, a pulse frequency of 7 kHz, and a resolution of 0.5 µm. The laser power was varied from 100 to 300 mW to adjust the degree of oxidation, and the corresponding samples were named as Mo_2_C‐P (0 mW), Mo_2_C‐100 (100 mW), Mo_2_C‐200 (200 mW), and Mo_2_C‐300 (300 mW) throughout the study. The fabricated nanoclusters benefit from forming multiple discrete Schottky heterojunctions, fast charge carrier transportation through the network, and more catalytic activities. The superior NH_3_ sensing performance of the chemiresistive sensor is implemented for food spoilage monitoring.

### 2.1 Material Characterizations

**Figure 2:**
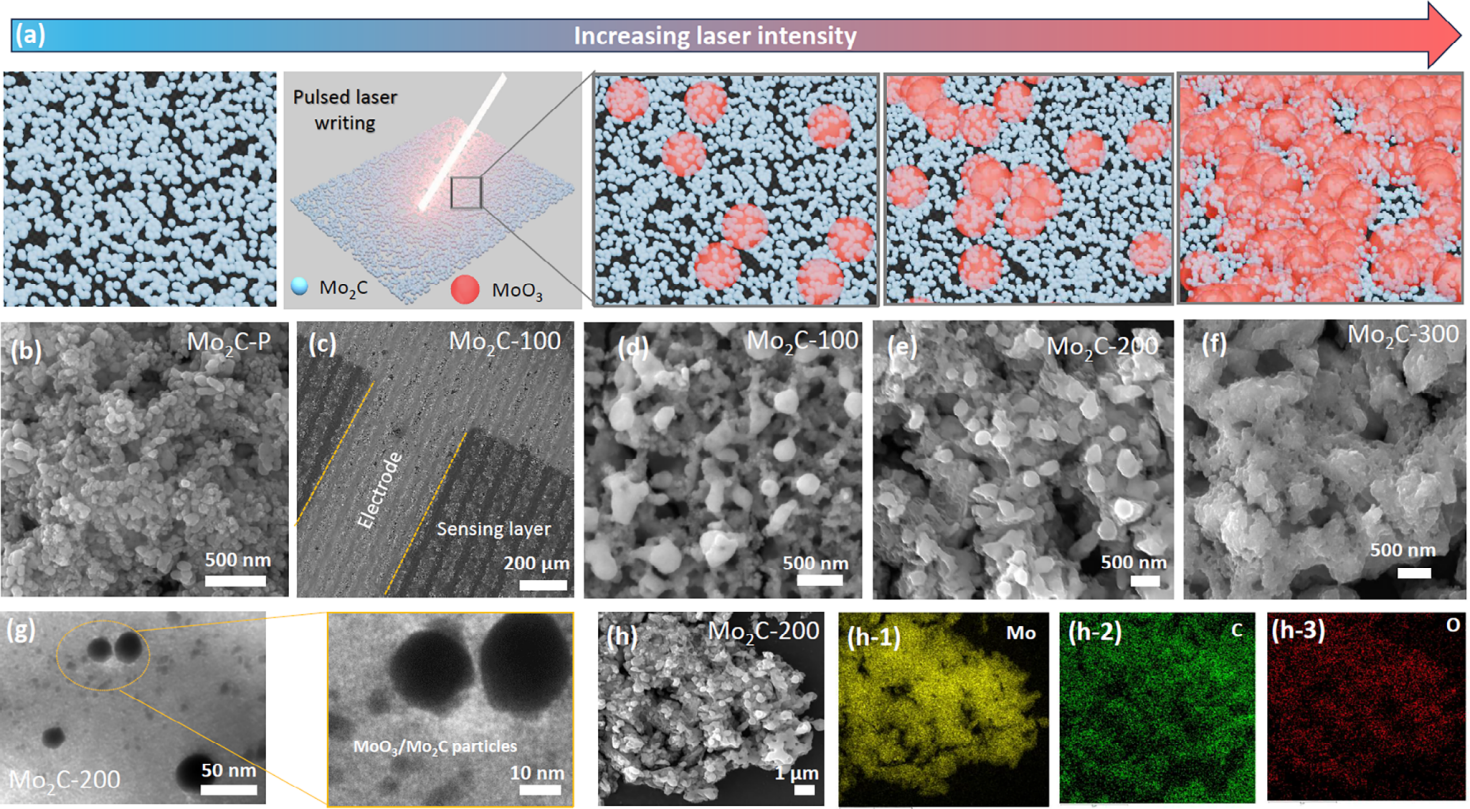
Morphological characterizations of materials by scanning electron microscopy (SEM). a) Synthesis flow of laser‐assisted Mo_2_C‐derived oxide. b) SEM image of pure Mo_2_C nanoparticles. c,d) SEM image of the fabricated device and Mo_2_C‐100 sample at higher magnification. e,f) SEM images of Mo_2_C‐200 and Mo_2_C‐300 samples. g) TEM micrograph of Mo_2_C‐200 sensing material, which represents the close interface between MoO_3_ nanoclusters on Mo_2_C nanoparticles. h) SEM and EDXanalysis of the Mo_2_C‐200 sample, where (h‐1) distribution of Mo, (h‐2) C, and (h‐3) O in the Mo_2_C sample after the laser treatment at 200 mW power. h‐1, h‐2, and h‐3 have the same scale bar as h.

As depicted in **Figure** **2**a, the carburized Mo_2_C nanoparticles underwent laser treatment to induce a patterned controlled oxidation in the material with associated scanning electron microscopy (SEM) images corresponding to each step (Figure 2b–f). The oxidation process was carried out using laser direct writing of the material layer on a SiO_2_ substrate. The laser beam with a scanned speed of 50 mm s^−1^ induces the multiphoton nonlinear adsorption and ionization to break the chemical bonds (Mo─C) and form MoO_3_ oxide from Mo_2_C nanoparticles.^[^ ^36^ ^]^ Figure 2b represents the SEM image of pristine Mo_2_C material, where Mo_2_C nanoparticles are uniformly deposited on the substrate, showing uniform and porous surface distribution. The nanosized Mo_2_C particles and porous distribution facilitate more gaseous analyte adsorption and gas diffusion than the 2D sheets of Mo_2_C MXene material.^[^ ^10^, ^11^, ^38^ ^]^ The size of the Mo_2_C clustered nanoparticles was estimated from the SEM results (Figure S1a, Supporting Information) using ImageJ software to be ≈61.1 nm (Figure S1b, Supporting Information), although a few clustered nanospheres reached in size of hundreds of nanometers. The controlled laser‐assisted oxidation formed a well‐patterned distribution of MoO_3_/Mo_2_C throughout the substrate (Figure 2c). In contrast to other traditional oxidation routes like solution oxidation and microwave heating, this technique offers the desired distribution of oxide in the sensing layer, and the extent of oxidation can be easily controlled. The morphology of the Mo_2_C nanoparticles was well maintained after laser treatment at a certain laser power, as clearly shown in Figure 2d–f. The distribution of MoO_3_ on Mo_2_C was also visualized using a low‐voltage transmission electron microscopy (TEM), as shown in Figure 2g. The spherical oxide and Mo_2_C particles are visible with a significant size variation in the TEM images. The contrast difference in TEM images also suggests the coexistence of both Mo_2_C and MoO_3,_ which is further validated by X‐ray diffraction (XRD) and Raman analysis. Both the MoO_3_/Mo_2_C materials formed an intimate connection at the interface, resulting in the formation of discrete Schottky junctions that surely modulate the charge carrier concentration and are beneficial for gas sensing (Figure 2g). The SEM images and energy dispersive X‐ray spectroscopy (EDX) mapping and spectra of the laser‐treated sample (Mo_2_C‐200) corresponding to 200 mW laser power are presented to understand the transformation in the Mo_2_Cmaterial (Figure 2h(1–3) and S1S1c in Supporting Information). During the laser treatment, MoO_3_ was formed on the surface of Mo_2_C nanoparticles, acting as a template nucleus and resulting in bumpy nanospheres of MoO_3_ (Figure 2d). With the increase in power, the growth of MoO_3_ clusters has gradually increased and developed a continuous layer upon the Mo_2_C nanoparticles, as observed from the SEM results in Figure 2f. These structural analyses revealed that the laser treatment of Mo_2_C nanoparticles formed heterogeneous structures with MoO_3_ oxide nanoclusters, which will enable the chemisorption and further diffusion of oxygenated species, maximizing the electron depletion region for improved sensing performance.^[^ ^11^, ^38^ ^]^

**Figure 3:**
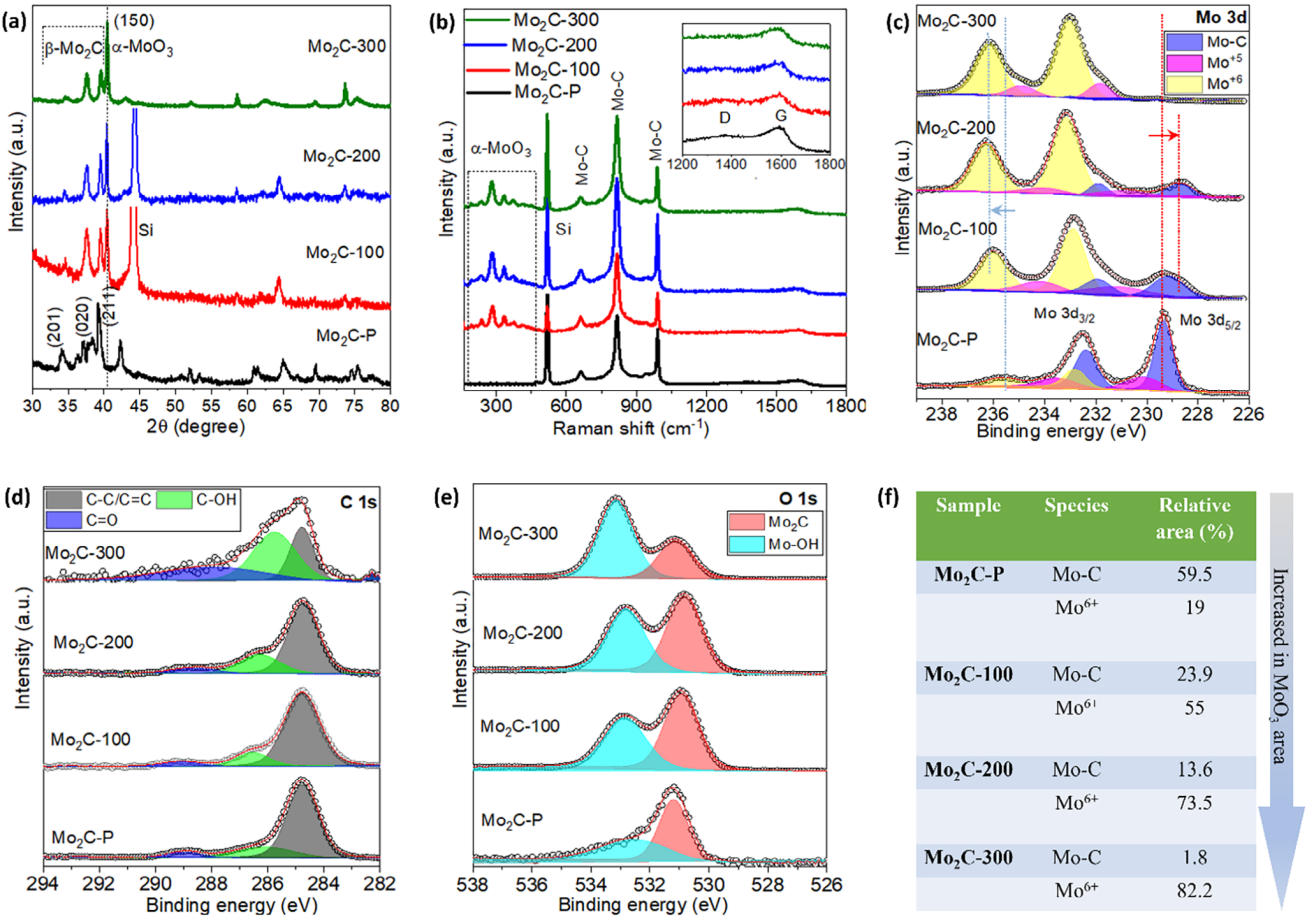
Structural and chemical characterizations of materials. a) XRD spectra. b) Raman spectra. c–e) XPS analysis of laser‐assisted Mo_2_C‐derived oxide samples. f) Calculation of the change in relative area of Mo‐C and MoO_3_ (Mo^+6^) in the samples after the laser writing from the Mo 3d XPS spectral analysis.

The strong XRD peaks originating from β‐Mo_2_C, and α‐MoO_3_ are visualized in **Figure** **3**a. These results indicate that the orthorhombic phase of β‐Mo_2_C (JCPDS 01‐077‐0720) and the orthorhombic phase of α‐MoO_3_ (JCPDS No. 80–950) are major phases in the heterostructure after the laser‐assisted controlled oxidation of pure Mo_2_C material. For β‐Mo_2_C, main peaks were observed at 34.5°, 37.9°, and 39.3°, which correspond to the (201), (020), and (211) lattice planes.^[^ ^10^, ^39^ ^]^ Furthermore, we qualitatively examined the trend of peak intensity in the XRD patterns before and after the oxidation treatment at different laser powers. As the laser power increases from 0 to 300 mW, the peak intensity of α‐ MoO_3_ (150) phase increases, and the characteristic peaks of β‐Mo_2_C gradually decrease, which indicates an increased content of oxide after the laser treatment.^[^ ^40^, ^41^, ^42^ ^]^ The change in intensities of two different phases (α‐ MoO_3_ and β‐Mo_2_C) at the different laser powers indicates the formation of a MoO_3_/Mo_2_C heterojunction.^[^ ^40^ ^]^

The Raman spectra in Figure 3b show the coexistence of fingerprint peaks corresponding to both α‐MoO_3_ and β‐Mo_2_C in the laser‐treated samples. The samples displayed two peaks at 819 and 999 cm^−1^ belong to β‐Mo_2_C, and two other broad peaks at ∼1350 and ∼1580 cm^−1^ represent the C phase in the material (inset of Figure 3b).^[^ ^43^, ^44^ ^]^ The lower spectral region Raman modes corresponding to bending and scissoring modes of Mo_3_─O were observed at 339 and 377 cm^−1^, respectively, and the peaks at 248 and 290 cm^−1^ are twisting and wagging modes of vibration, respectively, representing the formation of α‐MoO_3_ in the heterostructure material.^[^ ^45^, ^46^ ^]^ The D and G peak intensity decreases with the increase of α‐MoO_3_ content in the material, as shown in the inset of Figure 3b.

The change in the surface chemical composition and electronic states of Mo_2_C before and after laser treatment was analyzed by X‐ray photoelectron spectroscopy (XPS) measurements (Figure 3c–e). The Mo 3d spectra of samples (Figure 3c) are divided into three main groups, which are fitted into three pairs of peaks denoted to the Mo─C, Mo^+5^, and Mo^+6^ states in the material. The position of the peaks and the corresponding area are summarized in Table S1 (Supporting Information). Most studies claimed that decreased dominance of the Mo─C peaks and increased dominance of higher valence states (Mo^+5^ and Mo^+6^) in laser‐treated samples compared to the Mo_2_C‐P sample resulted from the controlled oxidation in the material.^[^ ^43^, ^44^ ^]^ With this hypothesis, Mo─C peaks red‐shifted (≈0.6 eV) and Mo^+6^ peaks blue‐shifted (≈0.7 eV) in laser‐treated samples compared with the peaks in Mo_2_C‐P, attributed to an electron transfer tendency from MoO_3_ to Mo_2_C in the heterojunction, confirming the successful synthesis of the MoO_3_/Mo_2_C composite.^[^ ^44^, ^47^ ^]^ Mark that the highest area of Mo^+6^ and vanished Mo‐C peaks in the Mo_2_C‐300 sample confirm the maximum surface coverage with the MoO_3_ during the laser treatment. Additionally, deconvoluted peaks in C1 s spectra of C─C/C═C, C─OH, and C═O are derived from Mo_2_C and MoO_3_ (Figure 3d). It was evident that the area of fitting peaks was significantly varying for the samples; the C─OH and C═O peaks area from the Mo_2_C‐100 to Mo_2_C‐300 samples increased, signifying that surface Mo_2_C has been converted into MoO_3_ with the increase in laser power.^[^ ^44^, ^47^ ^]^ The O 1s spectra in Figure 3e support the gradual increase of the Mo─OH peaks and a decrease in the Mo_2_C peak with the higher laser power in the samples. Most importantly, the surface oxygen or oxygen vacancy concentration exhibits a trend of increasing with the increase of laser power, which is due to more defects, catalytic and stronger ionization effect of MoO_3_, leading to improved surface interaction sites in the material (Figure 3e).^[^ ^11^, ^40^ ^]^ The increase of surface oxygen content substantially enhanced the oxidation ability to reducing gases like NH_3_, and faster adsorption and desorption of target gases on sensitive materials, which is crucial for improving gas sensing performance.^[^ ^11^, ^40^ ^]^ Moreover, XPS results indicated that a selected part of the Mo_2_C was converted into MoO_3_ and which can be tuned by increasing the laser power (Figure 3f), and the existence of MoO_3_/Mo_2_C heterojunction, consistent with the structural analysis (Figure 3a,b). The heterostructure formation is capable of enhancing the sensing performance of the Mo_2_C material by modulating electronic structures.^[^ ^38^ ^]^

### 2.2 Gas Sensing Study of Mo_2_C Gas Sensors

**Figure 4:**
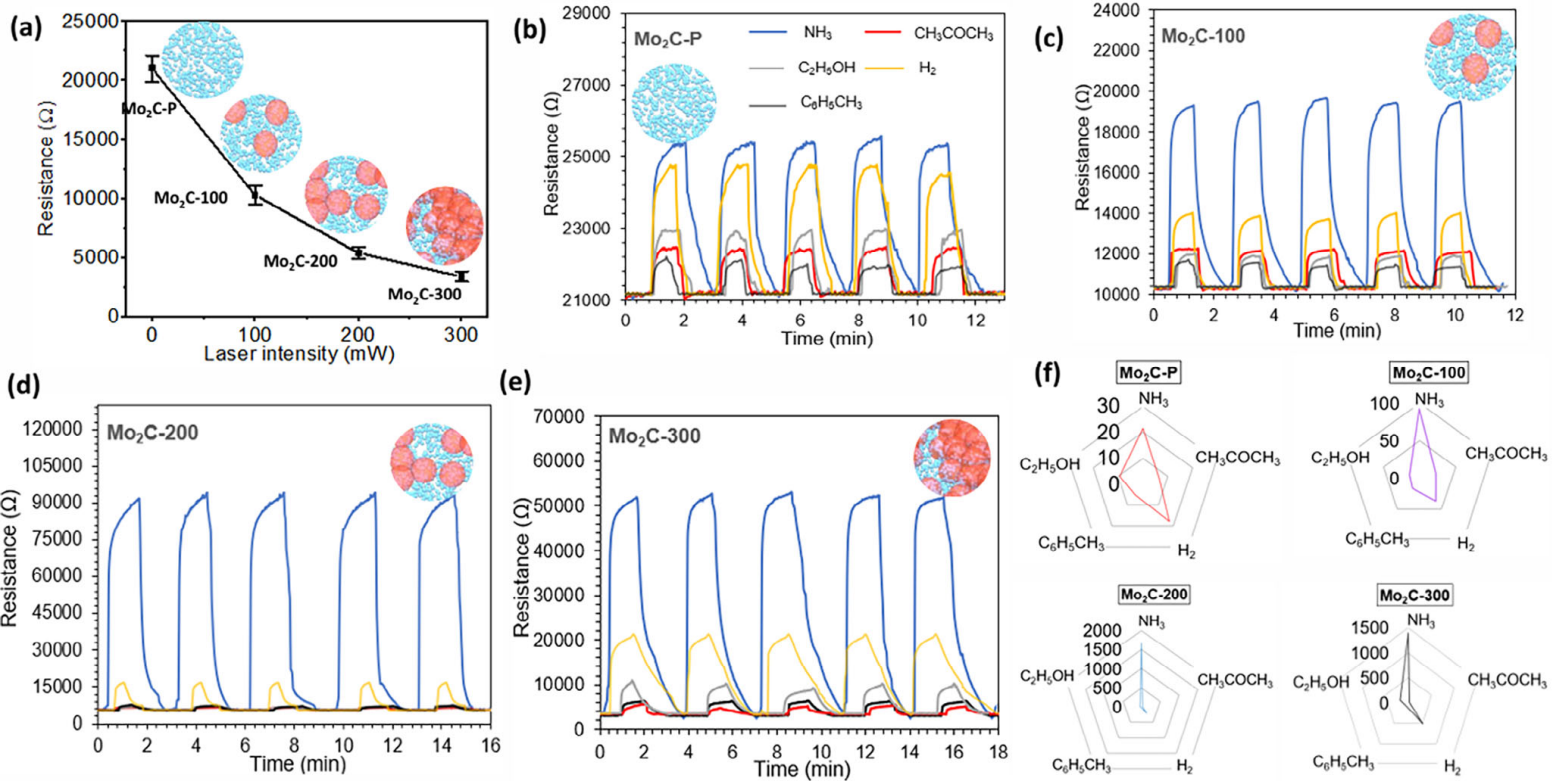
NH_3_ selectivity study of pure and laser‐treated Mo_2_C sensors. a) Change in baseline resistance with laser intensity. b–e) selectivity response curves of resistances of the Mo_2_C‐P, Mo_2_C‐100, Mo_2_C‐200, and Mo_2_C‐300 sensing devices for different gases and VOCs at room temperature and 63 ± 2% RH. f) Radar plot of the response of sensing devices toward different gases over the NH_3_ gas.

Room temperature gas sensors contribute to low power consumption and the design of simple and portable real‐time gas sensing systems. Here, Mo_2_C‐based gas sensing devices were tested in a two‐channel gas sensor test system at room temperature and 63 ± 2% RH. First, to understand the effect of different laser powers on the oxidation of Mo_2_C material, the change in resistance was observed. The resistance of the Mo_2_C‐100, Mo_2_C‐200, and Mo_2_C‐300 sensor devices decreased with increasing power of the laser beam, indicating the p‐type semiconducting characteristic of the sensing devices.^[^ ^10^, ^11^ ^]^ The laser treatment led to controlled oxidation in the material, which significantly changed the concentration of charge carriers in the sensing channel and subsequently decreased baseline resistance **Figure** **4**a. Apart from this, from the dynamic sensing curves, the resistances of the Mo_2_C‐P, Mo_2_C‐100, Mo_2_C‐200, and Mo_2_C‐300 sensing devices increased in the exposure to different reducing gases or VOCs at ambient temperature (Figure 4b–e), indicating a positive gas response behavior. The response of each Mo_2_C‐based gas sensor toward various gases or VOCs is summarized in Table S2 (Supporting Information). In contrast, partial oxidation of carburized Mo_2_C material by traditional thermal treatment routes either decreases the response or increases the operating temperature of the sensors.^[^ ^31^, ^35^ ^]^ The laser‐assisted controlled oxidized Mo_2_C material exhibited excellent sensitivity at room temperature and 63 ± 2% RH (Table S2, Supporting Information).

The selectivity of each sensor was examined by plotting the response values corresponding to the gases in the form of radar plots in Figure 4f. The Mo_2_C‐200 sensor showed outstanding sensitivity and the highest selectivity toward NH_3_. The pure Mo_2_C sensor (Mo_2_C‐P) showed good response values toward all the interfering gases and VOCs, attributed to the highly surface and edge reactivity of Mo_2_C, but the extremely poor selectivity toward NH_3_ gas. The laser‐treated sensors (Mo_2_C‐100 and Mo_2_C‐300) showed a similar selectivity trend of response toward NH_3_ increases and H_2_ decreases. Notably, higher laser intensity on Mo_2_C (Mo_2_C‐300) induced a substantial decrease in NH_3_ response. After a certain point, oxidation of the Mo_2_C material could lead to an increase in the activation energy to interact with the NH_3_ analytes and result in a reduced response value at room temperature and 63 ± 2% RH. However, the selectivity toward NH_3_ was intact in laser‐assisted oxidized sensors (Figure 4f). The great selectivity of Mo_2_C‐200 can originate from the formation of the appropriate amount of oxide functionalization and internal schottky junctions. The proven fact is that high charge density of NH_3_ analytes delivers more potential to shift the barrier height compared to the other gases and VOCs.^[^ ^48^ ^]^ Beside this, selectivity toward NH_3_ gas is attributed to NH_3_ catalytic activity of Mo_2_C in reduction, hydrogenation and nitrogen precipitation reactions.^[^ ^11^ ^]^

Clearly, laser‐assisted patterned MoO_3_/Mo_2_C sensors showed excellent sensitivity and selectivity characteristics compared to the pristine one. The oxidation at higher laser power (300 mW) induces excess oxidation of Mo_2_C, leading to a thick coverage of MoO_3_ oxide (Figure 2f), which hinders the interaction between the gas molecule and the Mo_2_C channel. On the other hand, the Mo_2_C‐100 sensor with less oxide functionality and reactive centers for the NH_3_ adsorption eventually shows less response value (Figure 3c,f). Therefore, the Mo_2_C‐200 sensor was the best‐performing sensor and was chosen for further studies.

**Figure 5:**
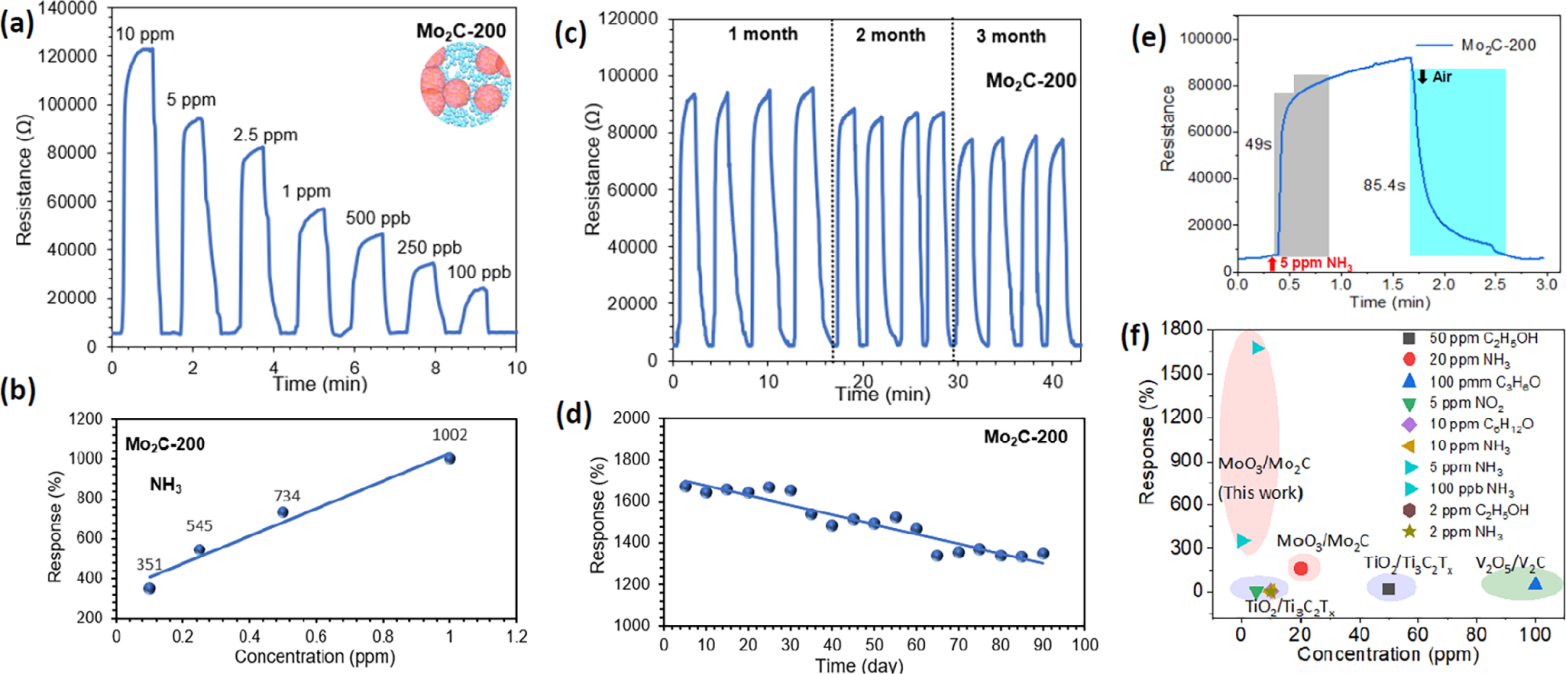
Gas sensing characteristics of the Mo_2_C‐200 sensor. a) transient sensing curve of the Mo_2_C‐200 sensor toward a range of NH_3_ gas concentrations. b) Linear fitting curve response versus concentration of the Mo_2_C‐200 sensor. c,d) long‐term stability behavior and corresponding response fluctuation of the Mo_2_C‐200. e) Response/recovery characteristics of the Mo_2_C‐200 sensor for 5 ppm of NH_3_ gas at room temperature and 63 ± 2% RH. f) Comparison of the state of the art of oxide or oxidized/TMC‐based gas sensors.

The transient gas sensing behavior and linear fitting results of the Mo_2_C‐P, Mo_2_C‐100, Mo_2_C‐200, and Mo_2_C‐300 sensors at a wide range of NH_3_ concentrations from 10 part per million (ppm) to 100 ppb were tested at room temperature and 63 ± 2% RH (**Figure** **5**a,b; Figure S2, Supporting Information). The responses of the sensors were increased in varying orders of magnitude with the increasing concentration of NH_3_ gas. Certainly, the pure Mo_2_C sensor showed a very slight change in resistance below 0.5 ppm NH_3_ concentration at room temperature and 63 ± 2% RH (Figure S2, Supporting Information). However, an excellent NH_3_ sensing performance was achieved in the Mo_2_C‐200 sensor with responses of 2214% to 10 ppm and 351% even to 0.1 ppm of NH_3_ (Figure 5b). The response of the Mo_2_C‐200 sensor corresponding to the lowest tested concentration (351%/100 ppb) was 245.4, 72.6, and 1.15 times higher than those of Mo_2_C‐P, Mo_2_C‐100, and Mo_2_C‐300 sensors, respectively (Figure S2, Supporting Information). All of the sensors have good linearity with R^2^ > 0.967 (Figure 5b; Figure S2, Supporting Information), following the Langmuir model.^[^ ^49^ ^]^ The Mo_2_C‐200 sensor showed a lower detection limit (LOD) of 29 ppb calculated from the improved IUPAC method.^[^ ^50^ ^]^ The long‐term stability test of Mo_2_C‐based sensors is worthwhile due to the good physical and chemical stability of TMC materials. As anticipated, the response of (Mo_2_C‐P) to 5 ppm of NH_3_ gas decreases abruptly after 5 days of exposure to ambient air and then decreases to ≈1 after 30 days, demonstrating that the Mo_2_C‐P sensor is out of operation (Figure S3, Supporting Information). On the other hand, the response of the Mo_2_C‐200 sensor toward 5 ppm of NH_3_ gas decreases slowly from ≈1675% to 1653% after getting exposed to air for 30 days (Figure 5c), and then the response declines to ≈1347% after 80 days (Figure 5c,d). Additionally, the response of other laser‐treated sensors (Mo_2_C‐100, Mo_2_C‐300) to 5 ppm of NH_3_ also decreased slowly after exposure to air for 90 days (Figure S4, Supporting Information). Moreover, the Mo_2_C‐200 sensor showed no obvious variation in the dynamic response and recovery process during 4 cycles to 5 ppm NH_3_ after exposure to air for 30‐day intervals at room temperature and 63 ± 2% RH (Figure 5d). The room temperature response to 5 ppm NH_3_ of the Mo_2_C‐200 sensor was ≈1653%, ≈1470%, and ≈1337% after exposure to air for 1, 2, and 3 months, respectively, signifying good reproducibility. Moreover, the laser‐treated Mo_2_C sensors have comparatively shorter response and recovery times than the pure Mo_2_C sensor, due to the higher capacity for oxygen adsorption and formation of discrete Schottky junctions and a wider depletion region, which permits faster electron mobility (Table S3, Supporting Information).^[^ ^10^, ^11^, ^20^, ^31^ ^]^ Notably, the Mo_2_C‐200 sensor displayed the highest response and shortest response and recovery times (49 and 85.4 s) than the other sensors (Figure 5e).

Our experimental results indicated that laser‐assisted controlled oxidized Mo_2_C material achieved benchmark‐level performance compared to other state‐of‐the‐art partially oxidized gas sensing layers at their optimum operating temperature (Figure 5f). The Mo_2_C‐200 sensor showed far higher responses to very low concentrations of NH_3_ at room temperature and 63 ± 2% RH. In particular, controlled oxidation in Mo_2_C material, using other routes, vanishes its room‐temperature gas sensing properties and requires a higher operating temperature to respond to gases. The high responses to ppb‐level NH_3_ are also compared with previously reported literature in Table S4 (Supporting Information), indicating that laser‐assisted controlled oxidation of Mo_2_C has superior NH_3_ sensing performance. The sensing capabilities of the Mo_2_C‐200 sensor are superior to those of other pure and oxidized TMCs, such as MXene materials (Figure 5f).

### 2.3 Gas Sensing Mechanism

**Figure 6:**
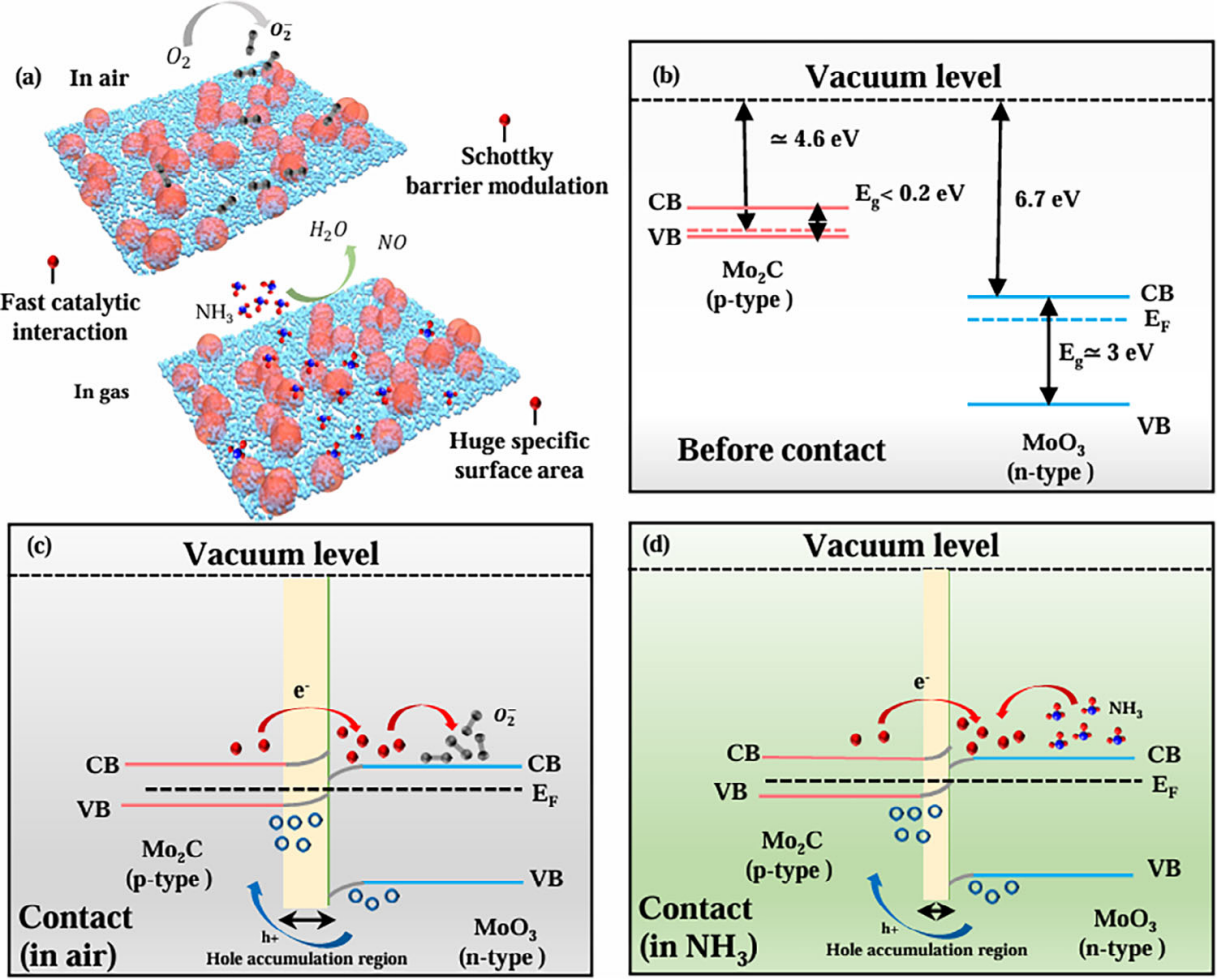
Gaseous interaction behavior and Schottky model for gas sensing mechanism. a) Schematic representation of the NH_3_ adsorption/desorption behavior on Mo_2_C‐200 sensing layer, b–d) discrete Schottky junction (S—S) between the Mo_2_C nanoparticles and MoO_3_ nanospheres (b) before contact, (c) after contact in air ambient, and (d) after contact in NH_3_ atmosphere.

The gas sensing mechanism of Mo_2_C‐derived MoO_3_ is dependent on the catalytic activities of the functionalized surface and discrete Schottky junctions between the materials (**Figure** **6**a). In general, bulk Mo_2_C has metallic characteristics, but high aspect ratio of carburized Mo_2_C nanoparticles and defective sites interactions with the environmental oxygen species, resulting in a small semiconducting bandgap and p‐type sensing response.^[^ ^10^, ^11^, ^12^, ^51^ ^]^ In this, Mo_2_C nanoparticles showed a positive response toward reducing NH_3_ gas.^[^ ^10^ ^]^ Further, laser treatment formed an oxide‐decorated surface on the top of the p‐type Mo_2_C channel, significantly changing the free charge carrier concentration by forming the discrete Schottky junctions. On the other hand, low formation energy and the occurrence of anion vacancies in MoO_3_ lead to an n‐type semiconducting material.^[^ ^52^ ^]^ In contrast, the sensors showed p‐type gas sensing response attributed to the carrier transport through the Mo_2_C conducting channel, thus holes proved the majority carriers and determined the type of sensing response in all sensors.^[^ ^11^ ^]^ In laser‐assisted functionalized sensors, MoO_3_ nanoclusters adsorb environmental oxygen and result in trapped electrons, which consequently decrease the resistance of the sensing layer (Figure 4a).^[^ ^11^ ^]^

Previous literature envisaged that metallic Mo_2_C is a narrow band gap (Eg < 0.2 eV) material,^[^ ^11^, ^12^, ^51^, ^53^ ^]^ with a work function value of ≈4.6, while MoO_3_ has a high band gap (Eg  =  3 eV)^[^ ^52^, ^54^ ^]^ with an electron affinity value of 6.7 eV. Figure 6b,c shows the band diagram of MoO_3_ oxide functionalized Mo_2_C before and after the formation of a Schottky junction. Due to the work function difference, free charge carriers will be transferred from the valence band of Mo_2_C to the conduction band of MoO_3_ oxide nanoclusters, resulting in a huge change in baseline resistance of the sensors.^[^ ^11^ ^]^ The extreme band offset flows electrons spontaneously, and the electron‐hole recombination continues until the Fermi level is reached at the equilibrium, resulting in a wide band bending at the interface (Figure 6c).^[^ ^52^ ^]^ The electron transfer from p‐type Mo_2_C not only accumulated the free hole carriers but also moved the Fermi level to show the strong p‐type behavior. This process also leads to the increased electron concentration on the contact surface of MoO_3_ nanoclusters (electron accumulation), providing increased capacity to adsorb the oxygen species. In the air ambient, adsorbed oxygen anion species trap the electrons from the MoO_3_ nanoclusters, and to maintain the Fermi level, Mo_2_C further transfers the electrons to MoO_3,_ resulting in an increased width of the hole accumulation layer, and a surface potential (qV_b‐air_). Whereas, in the presence of reducing NH_3_ gas, electrons injected back to the MoO_3_ surface inhibit electron transfer from Mo_2_C to MoO_3_. Therefore, the width of the hole accumulation layer decreased, as well as the surface potential (qV_b‐NH3_), leading to a larger increment in channel resistance (Figure 6d). This procedure ultimately reduces the barrier height (qφ_b‐NH3_) of the p‐n junction. The barrier height of a p‐n junction in the presence of air/NH_3_ gas can be expressed by Equation (1), where 𝐸𝑔 denotes the bandgap, 𝑞𝜒 represents the electron affinity of the semiconductor, and 𝑞𝛹 indicates the material's work function.^[^ ^55^, ^56^ ^]^

(1) $$q\varphi_{b-\mathrm{air}/NH_{3}}=E_{g-\mathrm{Mo}O_{3}}+q\chi_{\mathrm{Mo}O_{3}}-q\Psi_{Mo_{2}C-\mathrm{air}/NH_{3}}$$

Considering the alteration in the barrier height of p‐n heterojunction when exposed to air and NH_3_ environments, the response magnitude (RM) can be calculated using Equation (2).

(2) $$RM=\exp\left[\frac{\left(q\varphi_{b-NH_{3}\;}-\;q\varphi_{b-air}\right)}{kT}\right]$$

The p‐n heterojunction Schottky barrier decreases, and the mobility of generated charge carriers gets enhanced, leading to the low NH_3_ concentration detection.^[^ ^11^ ^]^ The laser‐assisted oxidation incorporated oxide nanoclusters in a well‐controlled manner, which enhances the catalytic activities of the sensing layer. The SEM results (Figure 2d–f) indicated that MoO_3_ nanoclusters are distributed across the Mo_2_C surface, indicating that laser‐assisted oxidation effectively inhibits MoO_3_ agglomeration and thus exposes more reactive sites. Additionally, electronic and chemical sensitization induced by MoO_3_ nanoclusters can increase the ionization capacity of oxygen, and the hydrogen precipitation effect of Mo_2_C enhances the reactivity of NH_3_, thereby synergistically enhancing the gas sensitivity.^[^ ^11^ ^]^

### 2.4 Food Spoilage Monitoring Application of Laser‐Treated Mo_2_C Sensor

**Figure 7:**
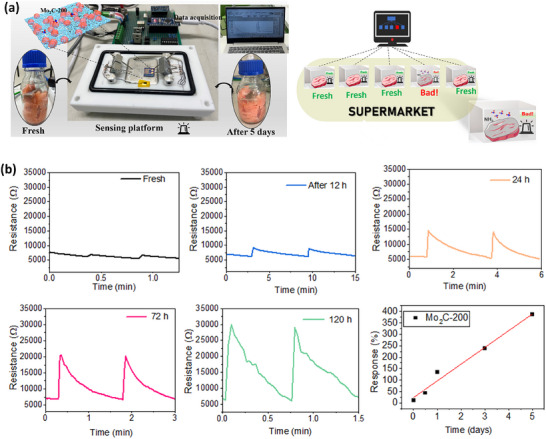
Food spoilage monitoring application of the Mo_2_C‐200 sensor at ambient conditions. a) Schematic figure of the sensing setup for the real‐time monitoring of fermentation in Atlantic salmon. b) response plots and linear fit of responses as a function of fermentation time of the Atlantic salmon fish.

To check the applicability of the Mo_2_C‐200 device as a practical NH_3_ gas sensor system for food quality monitoring application (**Figure** **7**a), an open gas sensing stage was designed which has two probes to connect the sensing device directly with computer terminals and a source measuring unit and fish bottle is connected to a syringe setup to flow the fermented air (Figure 7a; Figure S5 and Video S1, Supporting Information). We examined the real‐time NH_3_ gas sensing performance of the Mo_2_C‐200 sensor to monitor the freshness of fish. Specifically, the Mo_2_C‐200 sensor was exposed to the air collected from the closed bottle containing pieces of fish (Atlantic salmon), which was initially stored in a −18 °C refrigerator and then brought to RT (22 °C). The resistance of the device was measured at different fermentation intervals. The growth of microorganisms in fermented fish involves the urea and trimethylamine oxide into NH_3_.^[^ ^1^ ^]^ The sensor data were first measured from the fresh Salmon at room temperature (Figure 7b). After that, the air from a closed fermented fish bottle was collected using a syringe at different time intervals ranging from 12, 24, 72, and 120 h and injected near the sensor in an ambient atmosphere to validate the employment in real real‐time scenario, not in a controlled system at room temperature (Figure 7b). Additionally, the real‐time NH_3_ sensing characteristics from the fermented fish (fermentation time, 12 h) of the Mo_2_C‐200 sensor are recorded in a normal atmosphere to validate the real‐time applicability of the sensors (Video S2, Supporting Information). At zero fermentation hours, the sensor displayed no obvious responses, concluding that NH_3_ was not released from the fresh fish. The sensor response increased with the fermentation time and reached a response of 386% after 5 days of fermentation at room temperature. Although the sensor was not exposed to any additional airflow, and recovered from the ambient air after stopping the exposure to fermented air and reached the initial baseline resistance value in a very short time. The real‐time NH_3_ detection at different fish fermentation times showed a significant change in response from fresh to after 120 h of fish fermentation (Figure 7b), which indicates that the variation of the baseline resistance of the Mo_2_C‐200 device was completely caused by fish spoilage, confirming its extensive spoilage monitoring application prospect in the food industry.

## 3 Conclusion

In summary, we examined the laser‐assisted controlled synthesis of Mo_2_C‐derived MoO_3_ oxide and investigated the enhancement in NH_3_ sensing performance at room temperature. The formation of oxide nanoclusters on Mo_2_C resulted in a higher selectivity toward NH_3_ gas among five different gases or VOCs, mainly attributed to the intensity of the Schottky barrier sites in the heterostructure. Not only this, the NH_3_ selective properties of MoO_3_/Mo_2_C heterostructure are attributed to the highly oxidized surface of n‐p heterojunction, which was extremely favorable for the chemisorption of NH_3_ analytes. An optimally designed Mo_2_C‐200 chemiresistive sensor showed a high gas response (351%/100 ppb) toward the lowest tested NH_3_ concentration was ≈254 times higher than that of the pristine Mo_2_C sensor (Mo_2_C‐P), and attained a lower limit of detection with 29 ppb NH_3_ gas at room temperature and 63 ± 2% RH. The Mo_2_C‐based sensor also reported extraordinary long‐term stability with a response attenuation of 20% after 3 months of testing in ambient conditions. This new strategy of introducing controlled oxidation in TMCs material by laser‐assisted treatment improves the stability and selectivity of the target analyte at room temperature and 63 ± 2% RH. Further, the real‐time food spoilage monitoring application of the Mo_2_C‐200 sensor shows extensive potential in designing TMC‐based practical NH_3_ gas sensors. Based on the methodology, it also incorporates the role of laser power on Schottky barrier modulation and surface reactive sites. The simple laser technique can be implemented on other available TMC materials (V_2_C/Nb_2_C, etc.) surfaces to prepare a highly sensitive/selective gas sensing platform.

## 4 Experimental Section

### β‐Mo_2_C Synthesis

*β*‐Mo_2_C was developed using a temperature‐programmable carburization procedure in a tube furnace. 200 mg of ammonium molybdate ((NH_4_)_6_Mo_7_O_24_, Sigma‐Aldrich) as a Mo source was added in 20 mL of ethanol and 30 mL of deionized (DI) water. 0.2 gm urea as a carbon source has been added to the solution. The solution was then transferred to a polytetrafluoroethylene (PTFE) pressure round‐bottom flask and heated at 90 °C in an oil bath for 6 h. Later, the solution was dried in a vacuum oven, and the precipitated powder was placed in a tube furnace. The temperature was increased from room temperature to 550 °C with a ramping rate of 10 °C min^−1^ under an argon gas flow. Ar/H_2_ gas was injected and maintained for 3 h for carburization.

### β‐Mo_2_C Laser Direct Writing

First, 3 mg of *β*‐Mo_2_C was dispersed into 3 mL of ethanol/water (45 vol%) under continuous ultrasonication for 20 mins. A 20 µL of *β*‐Mo_2_C solution was spin‐coated onto a SiO_2_/Si substrate at 500 rpm of speed and then dried for 24 h at room temperature in N_2_ atmosphere. The diode‐pumped solid‐state Nd:YAG laser direct writing system with a wavelength of 532 nm, a pulse frequency of 7 kHz, and a resolution of 0.5 µm. An oil‐immersion objective lens (60×, NA 1.4, Olympus) focused the laser beam onto the *β*‐Mo_2_C film coated on SiO_2_/Si substrate while placed on a 3D piezoelectric stage. A 100 µm ^−1^s laser scanning speed and the laser intensities vary from 100 to 300 mW to write the patterned sensing layers on the substrate. The samples treated with different laser power were marked as Mo_2_C‐P (0 mW), Mo_2_C‐100 (100 mW), Mo_2_C‐200 (200 mW), and Mo_2_C‐300 (300 mW) throughout the study.

### Fabrication of Chemi Resistive Gas Sensors

Interdigitated top Au electrodes of 150 nm thickness were deposited on laser‐patterned Mo_2_C samples, using a Cu physical mask by electron beam evaporation technique. An annealing procedure was carried out in an Ar ambient at 100 °C using rapid thermal annealing (RTA) to stabilize the properties of fabricated devices in the external atmosphere.

### Material Characterization

SEM images were captured from FEI VERIOS 460L with an acceleration voltage of 10−20 kV. The elemental mapping and atomic% % were taken with an EDX detector (EDAX SDD Octane Super) attached along with the FEI VERIOS 460L. XRD spectra were measured using an X‐ray powder diffractometer (Rigaku3, SmartLab). Surface chemical composition analysis was studied with an XPS (Kratos, a Kratos Analytical Axis Supra instrument) spectrometer using a monochromatic Al Kα (1486.7 eV) excitation source. All XPS data were calibrated at 284.8 eV to the adventitious C 1s peak and fitted with CasaXPS software. Raman spectra were recorded using a Raman spectrometer (Witec Alpha 300R) with a 532 nm laser. TEM analysis was done by using a low‐voltage TEM (LVEM 25; energy 25 KeV) instrument.

### Gas Sensing Setup and Measurements

The gas‐sensing tests will be conducted in a home‐built Teflon‐sealed chamber at room temperature. The sensing behavior of devices will be measured using a source meter (Keysight 2601). The source bottle of NH_3_ gas contains 50 ppm NH_3_ gas in a background of synthetic air, with a purity of N6.0. Gas mixing was performed using a gas mixer setup comprising low‐flow mass flow controllers (ALICAT Scientific) and a mixing bottle. The overall volume of the sensing environment will be fixed at 65 ml/min. The sensing response of the sensors will be calculated as ((R_gas_‐R_air_)/R_air_) × 100 where R_gas_ and R_air_ are the measured resistances of the sensors in the exposure to test gases and dry air, respectively. The response time will be calculated as a 90% change in the resistance from air to gas.

## Conflict of Interest

The authors declare no conflict of interest.

## Author Contributions

R.B. and M.P. conceptualized and designed the experiments. R.B. fabricated the devices, carried out the materials characterizations, tested the gas sensing behavior, and drafted the manuscript. S.D. carried out the laser patterning part and helped with the materials characterizations and manuscript writing. M.P. supervised the research and provided financial support. All authors discussed the results, contributed to the manuscript corrections, and approved the final version of the manuscript.

## Supporting information



Supporting Information

Supplemental Video 1

Supplemental Video 2

## Data Availability

The data that support the findings of this study are available from the corresponding author upon reasonable request.
