# Supplementary material for: Laser‐Assisted Mo2C‐Derived Patterned Oxide for Highly Selective Room Temperature Ammonia Sensor for Food Spoilage Monitoring
Source: Small Methods. 2025 Sep 16;9(11):e01246. doi: 10.1002/smtd.202501246 (PMC12641371; doi:10.1002/smtd.202501246)
Supplement: Supplementary file 1 — Supporting Information [file SMTD-9-e01246-s001.docx]

Laser-assisted Mo_2_C-derived patterned oxide for highly selective room temperature ammonia sensor for food spoilage monitoring

*Radha Bhardwaj,^a,^ Sujit Deshmukh,^a^ Martin Pumera^a,b,c,d^*

^a^ Future Energy and Innovation Laboratory, Central European Institute of Technology, Brno University of Technology, Purkyňova 123, Brno, 61200, Czech Republic

^b^ Department of Medical Research, China Medical University Hospital, China Medical University, No. 91 Hsueh-Shih Road, Taichung, 40402, Taiwan

^c^ Department of Chemical and Biomolecular Engineering, Yonsei University, 50 Yonsei-ro, Seodaemun-gu, Seoul, 03722, Korea

^d^ IT4Innovations, VSB – Technical University of Ostrava, 17. listopadu 2172/15, Ostrava-Poruba 70800, Czech Republic


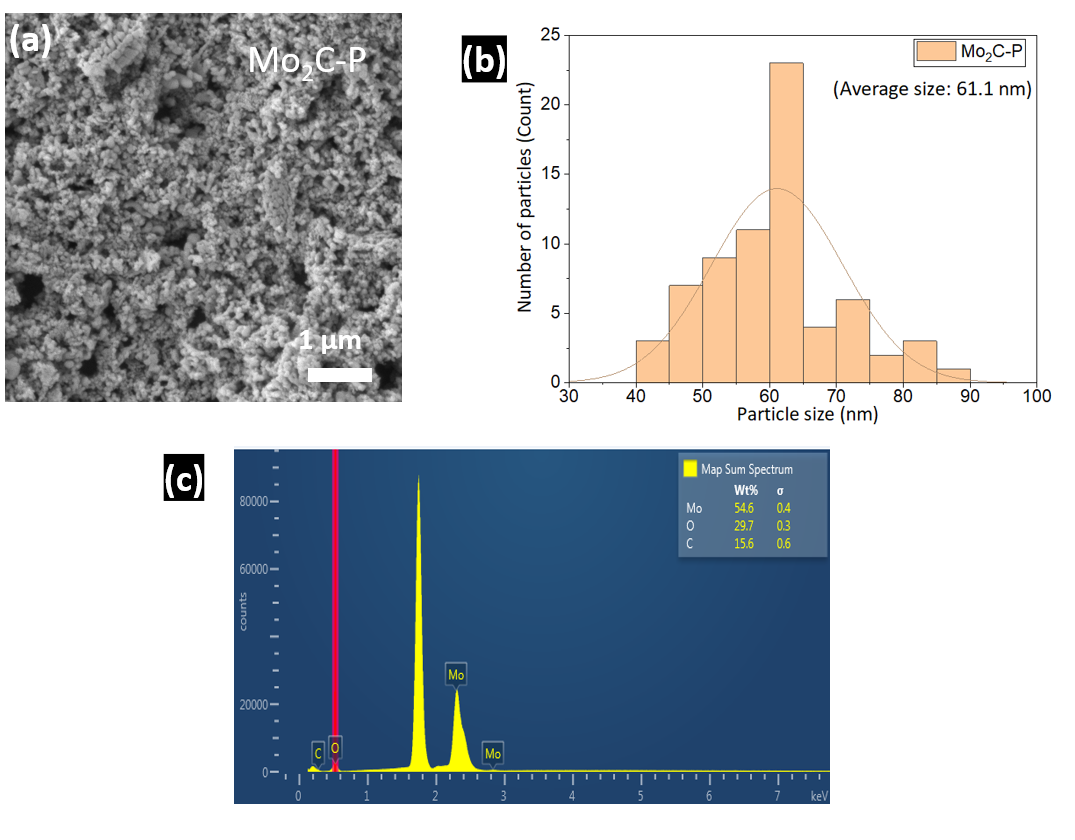


**Figure S1.** (a) Low-resolution SEM image of pure Mo_2_C nanoparticles, (b) size distribution of pure Mo_2_C nanoparticles calculated by using ImageJ software (c) EDX spectrum of Mo_2_C-200 sample.

**Table S1.** (a) binding energy and relative area estimation of the deconvoluted peaks of Mo 3d spectra.

| sample | Species | Binding energy (eV) | Area | Relative area (%) |
| --- | --- | --- | --- | --- |
| **Mo_2_C-P (Mo 3d_5/2_/Mo 3d_3/2_)** | Mo-C | 229.3/232.3 | 6487/4324 | 59.5 |
|  | Mo^5+^ | 230.1/233.5 | 2350/1567 | 21.5 |
|  | Mo^6+^ | 232.8/235.6 | 2047/1365 | 19 |
| **Mo_2_C-100 (Mo 3d_5/2_/Mo 3d_3/2_)** | Mo-C | 229.1/231.9 | 22935/15290 | 23.9 |
|  | Mo^5+^ | 231.1/234.1 | 20225/13483 | 21.1 |
|  | Mo^6+^ | 232.8/236 | 52657/35104 | 55 |
| **Mo_2_C-200 (Mo 3d_5/2_/Mo 3d_3/2_)** | Mo-C | 228.9/231.8 | 2031/1354 | 13.6 |
|  | Mo^5+^ | 231.2/234 | 1936/1290 | 12.9 |
|  | Mo^6+^ | 232.9/236.1 | 11008/7338 | 73.5 |
| **Mo_2_C-300 (Mo 3d_5/2_/Mo 3d_3/2_)** | Mo-C | 229.9/232.9 | 2544/1696 | 1.8 |
|  | Mo^5+^ | 231.8/234.9 | 22040/14693 | 16 |
|  | Mo^6+^ | 233/236.1 | 112413/74942 | 82.2 |

| **Sensor** | **NH_3_** | **H_2_** | **CH_3_COCH_3_** | **C_6_H_5_CH_3_** | **C_2_H_5_OH** |
| --- | --- | --- | --- | --- | --- |
| Mo_2_C-P | 21.4 | 17.6 | 6.6 | 5.2 | 9.5 |
| Mo_2_C-100 | 92.2 | 37.4 | 22.5 | 16.6 | 13.9 |
| Mo_2_C-200 | 1673.1 | 214.8 | 33.3 | 24 | 51.8 |
| Mo_2_C-300 | 1390 | 505.7 | 28.5 | 71.4 | 185.7 |

**Table S2.** Response (%) of different laser treated Mo_2_C MXene sensors towards different interfering gases and VOCs for 5 ppm of concentration at room temperature.

**
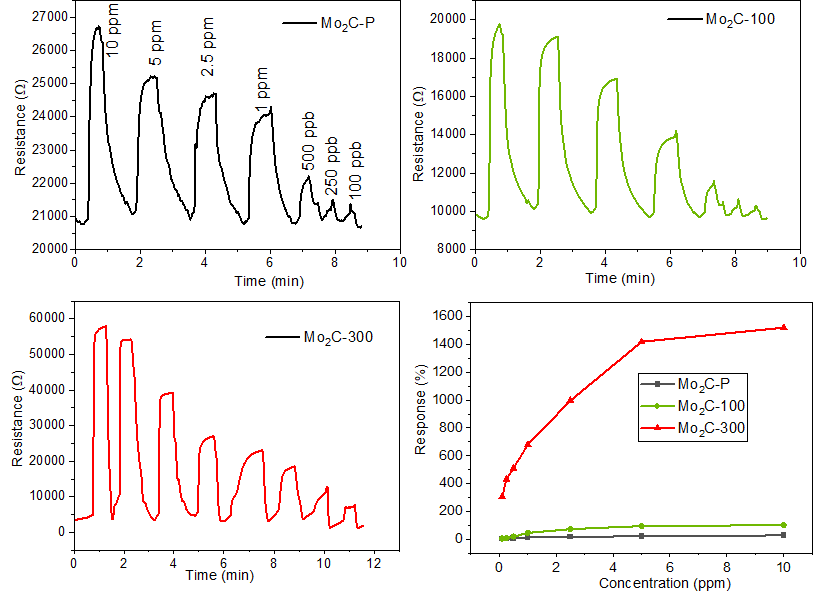
**

**Figure S2.** Transient characteristics and response behavior of Mo_2_C-P, Mo_2_C-100 and Mo_2_C-300 sensors at room-temperature based on the partially oxidized MXenes using different oxidation techniques.


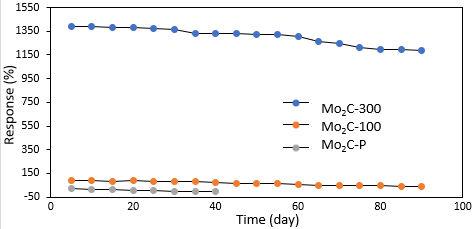


**Figure S3.** Long-term stability response fluctuation of the Mo_2_C-P, Mo_2_C-100, and Mo_2_C-300 sensors for 5 ppm NH_3_ at room temperature for 90 days with an interval of 5 days.


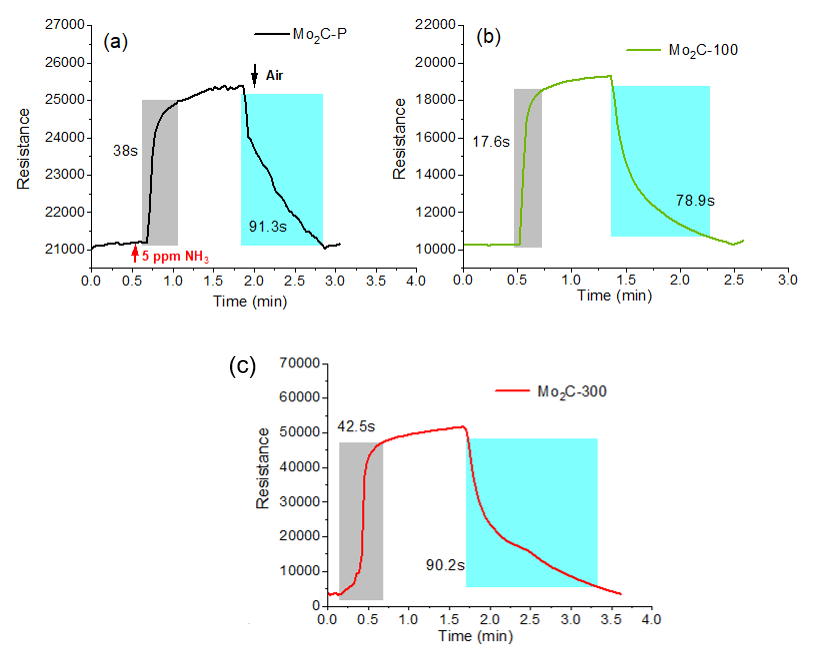


**Figure S4.** Response/ recovery time characteristics of Mo_2_C-P, Mo_2_C-100 and Mo_2_C-300 sensors for 5 ppm NH_3_ concentration at room temperature.

| **Sensor** | **Sensitivity (%) for 50 ppm** | **Sensitivity (%) for 100 ppb** | **Response/recovery time for 50 ppm NH_3_** | **Operating conditions** |
| --- | --- | --- | --- | --- |
| Mo_2_C-P | 21.4 | 1.4 | 38 s/ 91.3 s | 65% RH and 20 °C (RT) |
| Mo_2_C-100 | 92.2 | 4.8 | 17.6 s/78.9 s | 65% RH and 20 °C (RT) |
| Mo_2_C-200 | 1673.1 | 351 | 49 s/ 85.4 s | 65% RH and 20 °C (RT) |
| Mo_2_C-300 | 1390 | 304 | 43.5 s/90.2 s | 65% RH and 20 °C (RT) |

**Table S3.** Sensor-to-sensor variation in the gas sensing characteristics of different laser-treated Mo_2_C sensors towards NH_3_ gas.

**Table S4.** Sensing performance comparison of room-temperature gas sensors based on the oxide or oxidized/TMC materials based gas sensors using different oxidation techniques.

| Material | Oxide | Method | Target gas | Concentration (ppm) | Response | Operation temp. | Ref. |
| --- | --- | --- | --- | --- | --- | --- | --- |
| Mo_2_C | MoO_3_ | Hydrothermal | NH_3_ | 20 | ∼160% | RT | 11 |
| V_2_CT_x_ | V_2_O_5_ | Calcination | C_3_H_6_O | 100 | ∼45.3% | RT | 29 |
| Ti_3_C_2_T_x_ | TiO_2_ | Oxygen plasma | C_2_H_5_OH | 50 | 17.1% | RT | 35 |
| Ti_3_C_2_ | TiO_2_ | Solution method | NO_2_ | 5 | ∼1.17% | RT | 48 |
| Ti_3_C_2_T_x_ | TiO_2_ | Hydrothermal growth | C_6_H_12_O | 10 | 3.4% | RT | 31 |
| Ti_3_C_2_T_x_ | TiO_2_ | Solution mixing | NH_3_ | 10 | ∼3.1% | RT | 28 |
| Ti_3_C_2_T_x_ | TiO_2_ | Treatment dependent | NH_3_ | 2 | ∼2% | RT | 32 |
| Mo_2_C | MoO_3_ | Laser treatment (Mo_2_C-200) | NH_3_ | 5 | 1675% | RT | This work |
| Mo_2_C | MoO_3_ | Laser treatment (Mo_2_C-200) | NH_3_ | 0.1 | 351% | RT | This work |


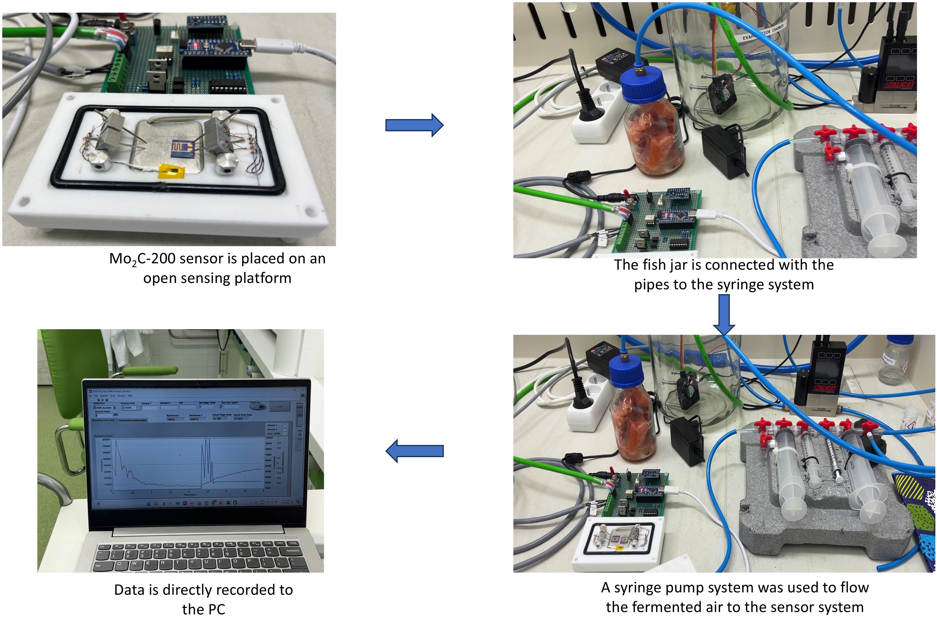


**Figure S5.** Food spoilage application setup with the fermented fish sample of the Mo_2_C-200 sensor at room temperature and ambient condition.
